# Are drivers of root-associated fungal community structure context specific?

**DOI:** 10.1038/s41396-019-0350-y

**Published:** 2019-01-28

**Authors:** A. Khuzaim Alzarhani, Dave R. Clark, Graham J. C. Underwood, Hilary Ford, T. E. Anne Cotton, Alex J. Dumbrell

**Affiliations:** 1 School of Biological Sciences, University of Essex, Wivenhoe Park, Colchester, Essex, CO4 3SQ UK; 2 School of Environment, Natural Resources and Geography, Thoday buildings, Bangor University, Bangor, LL57 2DG UK; 3 Present Address: Faculty of Science, Northern Border University, Arar, Saudi Arabia; 4 Present Address: Department of Animal and Plant Sciences, The University of Sheffield, Alfred Denny Building, Sheffield, SY S10 2TN UK

**Keywords:** Microbial ecology, Macroecology, Fungal ecology

## Abstract

The composition and structure of plant-root-associated fungal communities are determined by local abiotic and biotic conditions. However, the relative influence and identity of relationships to abiotic and biotic factors may differ across environmental and ecological contexts, and fungal functional groups. Thus, understanding which aspects of root-associated fungal community ecology generalise across contexts is the first step towards a more predictive framework. We investigated how the relative importance of biotic and abiotic factors scale across environmental and ecological contexts using high-throughput sequencing (*ca*. 55 M Illumina metabarcoding sequences) of >260 plant-root-associated fungal communities from six UK salt marshes across two geographic regions (South-East and North-West England) in winter and summer. Levels of root-associated fungal diversity were comparable with forests and temperate grasslands, quadrupling previous estimates of salt-marsh fungal diversity. Whilst abiotic variables were generally most important, a range of site- and spatial scale-specific abiotic and biotic drivers of diversity and community composition were observed. Consequently, predictive models of diversity trained on one site, extrapolated poorly to others. Fungal taxa from the same functional groups responded similarly to the specific drivers of diversity and composition. Thus site, spatial scale and functional group are key factors that, if accounted for, may lead to a more predictive understanding of fungal community ecology.

## Introduction

The identity, abundance, and number of different species contained within an ecosystem ultimately underpin all of that ecosystem’s functions [1–3]. Consequently, changes in community structure will have significant effects on ecosystem processes and functioning, and by extension, ecosystem services [4–6]. Therefore, elucidating the drivers of diversity and community structure is of paramount importance if we are to manage ecosystems in the face of continued environmental change. Plant-root-associated fungi are one such group known to significantly affect ecosystem processes and functions [7–12] as they influence individual plants and entire plant communities via a spectrum of plant–fungal interactions, ranging from highly phytobeneficial to highly phytodetrimental [13–16].

The development of molecular methods has significantly improved our understanding of how root-associated fungal communities are influenced by the environment [17–20] and studies are beginning to address this question over global scales [21–23]. A variety of biotic and abiotic drivers of fungal diversity and community composition have emerged, including variables related to plant diversity, identity and traits [24–26], edaphic variables such as pH, salinity and soil moisture [17, 22] and climatic variables such as seasonality and precipitation [19, 20, 26, 27]. However, previous studies have covered a range of conditions (e.g., different spatial/temporal scales, bioclimatic regions, habitat types etc.) and fungal functional groups, and the extent to which any identified drivers of community structure generalise across different studied systems remains unclear. For instance, the relative roles of biotic and abiotic factors in modulating fungal diversity is widely debated, with plant alpha- and beta-diversity, and plant identity [28–31], all cited as being significant drivers, whilst others studies find little, or no, effect of biotic variables in structuring fungal communities, with abiotic variables dominating [32–34]. Furthermore, studies have recorded differing ecological responses to environmental variables between fungal functional groups [35–38]. This lack of generalisable relationships between fungal communities and the biotic and abiotic conditions of a given study system, suggests that drivers of root-associated fungal community structure may be dependent on environmental (the suite of all interacting abiotic physical and chemical variables that influence a species) and/or ecological (the surrounding assemblage of all biotic interactions, in which a given species is embedded) contexts. Thus, while a single abiotic variable (e.g., pH) may be the main driver of root-associated fungal community composition within an ecosystem operating under one set of environmental and ecological contexts, it may not be the main driver in another, even if these two ecosystems ostensibly represent the same type of habitat. This is because subtle changes in one or more abiotic variable(s) may interact, and/or the strength and nature of species interactions within that ecosystem may change (e.g., dispersal limitation may cause local endemism [39] and thus which species can interact), respectively, leading to distinct environmental and ecological contexts influencing fungal species. This could then change the primary driver of fungal community composition from one abiotic variable to another, or between abiotic and biotic variables. Thus, models of fungal diversity and community structure in relation to environmental parameters are likely to generalise poorly to new contexts, resulting in poor estimation of fungal community structure in other ecosystems. However, how well models of root-associated fungal diversity and community structure established for a given environmental or ecological context generalise to others remains to be empirically tested.

To test for context-dependency in fungal community structure, an ideal model system would allow for large (spatial and temporal) replication within sites, but also across sites spanning biogeographic scales (here >100 km), in order to sample from a suite of environmental and ecological contexts. Additionally, sites should contain the same habitat in order to maximise comparability. Salt marshes are globally important, widespread habitats that provide valuable ecosystem services, including coastal protection [40] and carbon sequestration [41]. The diversity of root-associated fungi in salt marshes is under-explored, and these habitats are an ideal system in which to examine context-dependency as they contain distinct environmental and ecological contexts in relation to salt marsh zonation, are sufficiently large to allow extensive spatial replication, and are widespread around coastal areas, allowing for replication across biogeographic scales. The few existing studies of salt marsh mycobiomes suggest an important role for abiotic and biotic variables in structuring fungal communities [42–45], but the relative importance of these variables in driving diversity and community structure, and whether these generalise between salt marsh systems, or fungal functional groups is unclear (see [44]). Therefore, we studied the root-associated fungal communities from six UK salt marshes, spanning two geographically and floristically distinct regions, in both summer and winter. Importantly, few plant species were shared between regions, representing distinct ecological contexts; while within regions, each of the three sites differed in tidal exposure creating distinct environmental (physicochemical) contexts, allowing us to disentangle the importance of context within a series of comparable habitats. We quantified a range of biotic and abiotic factors that have previously been shown to influence root-associated fungal communities, and used them to model the richness and community composition of fungal taxa within different salt marshes, spatial scales, and functional groups. If the ecological drivers of root-associated fungal communities are context-specific, we hypothesise that:

H1: (a) The identity, direction and predictive power of biotic and abiotic drivers of root-associated fungal diversity will differ between sites and spatial scales, and consequently, (b) models of fungal richness will make inaccurate predictions when applied to other sites.

H2: The relative importance of biotic and abiotic drivers of root-associated fungal community structure will be consistent within, but not between, spatial scales and, biotic variables will be more important at larger spatial scales, reflecting the floristic dissimilarity at the regional level.

H3: Root-associated fungal taxa that share similar modelled responses to biotic and abiotic variables comprise clearly defined ecological groups (ecogroups) that in turn reflect the differing functional traits/attributes present within the fungal community.

## Materials and methods

### Study sites and sampling

**Fig. 1:**
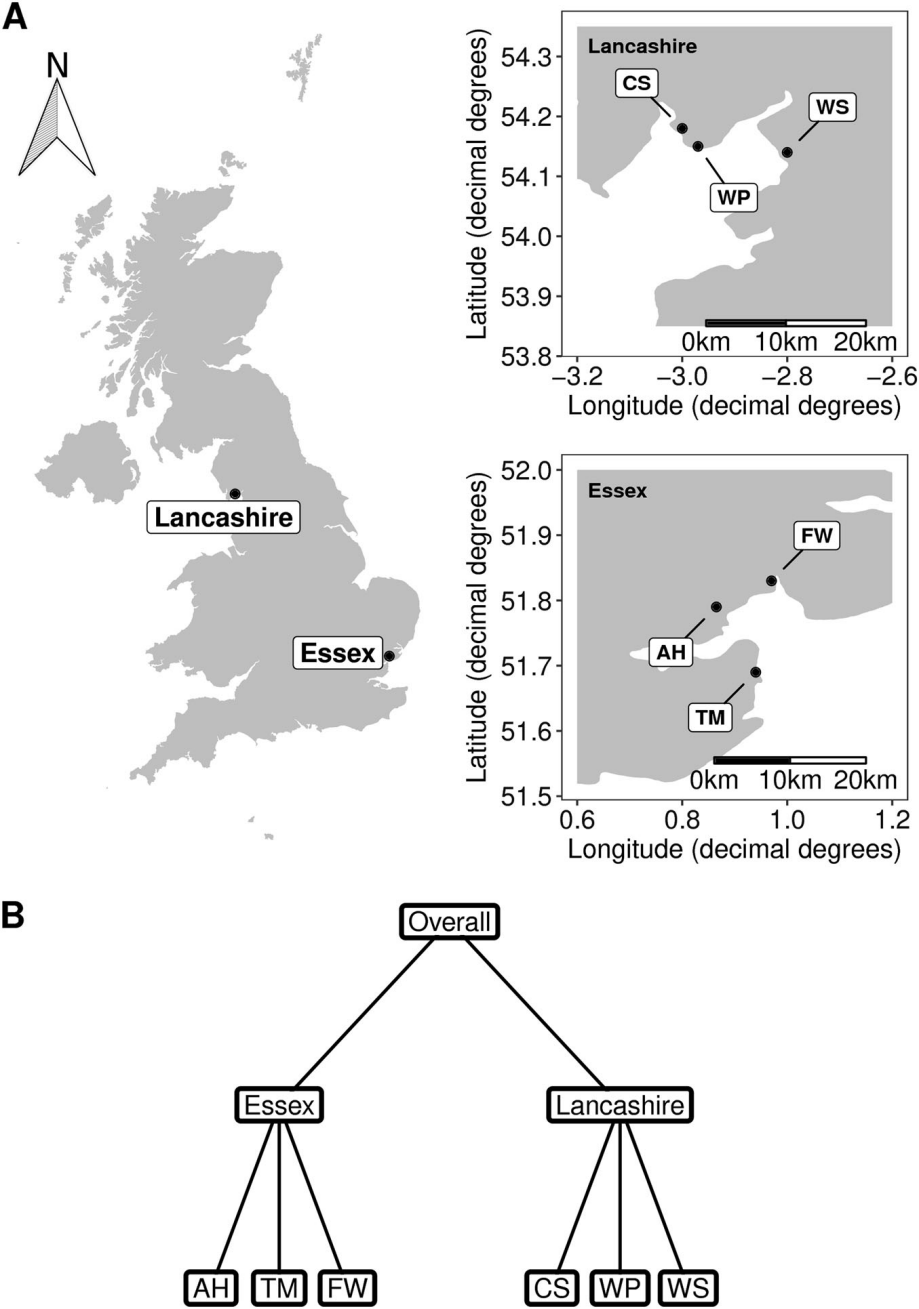
Sampling strategy. **a** UK map with sampling regions (left panel) and sampling sites (right panels). Site labels are as follows; AH Abbotts Hall, FW Fingringhoe Wick, TM Tillingham Marsh, CS Cartmel Sands, WP West Plain and WS Warton Sands. Coordinates are presented in WGS84 coordinate system. **b** Schematic of fungal OTU richness modelling approach. Models were conducted at the site level (bottom row), regional level (middle row) and with all data pooled (top row)

Sampling was conducted during winter (13/01/2013–05/02/2013) and summer (01/08/2013–16/09/2013) from three salt marsh sites in each of two regions, Essex (Abbotts Hall (AH), Fingringhoe Wick (FW) and Tillingham Marsh (TM)), and Lancashire (Cartmel Sands (CS), West Plain (WP) and Warton Sands (WS)), within the UK (Table 1 and Fig. 1a). A stratified random sampling design was used to place 22 quadrats in each marsh during each season, whilst maximising the range of spatial separation between samples (full details in ref. [46]). From each quadrat, a sediment core (5-cm diameter, 15-cm depth) was collected during each season (264 cores in total). Cores were transported from the field on dry ice before being frozen at −80 °C. Plant roots were extracted, washed and dried at 75 °C for 5 days, before being homogenised and stored at −80 °C for molecular analyses. Pore water was extracted from 40 g of sediment from each core by centrifugation at 15,000 rpm at 4 °C, and used for salinity and pH analyses (Table S2). The composition of the plant community in each quadrat was quantified as described previously [47] with the percentage cover of species, and above- and below-ground biomass measured (Table S2). All plant data are available via the Environmental Information Data Centre (EIDC) database: [https://catalogue.ceh.ac.uk/eidc/documents#term=CBESS&page=1].

**Table 1:** Full details of sampling locations and topological characteristics

Site	Region	Tidal exposure	Sediment type^a^	Number of samples sequenced^b^	Latitude	Longitude
Fingringhoe Wick (FW)	Essex	Minimal	Clay	(summer) 14 (winter) 17	51.83	0.97
Abbotts Hall (AH)	Essex	Intermediate	Clay	(summer) 22 (winter) 15	51.79	0.87
Tillingham Marsh (TM)	Essex	Severe	Clay	(summer) 22 (winter) 22	51.69	0.94
Cartmel Sands (CS)	Lancashire	Minimal	Sand	(summer) 22 (winter) 13	54.18	−3.00
West Plain (WP)	Lancashire	Intermediate	Sand	(summer) 21 (winter) 18	54.15	−2.97
Warton Sands (WS)	Lancashire	Severe	Sand	(summer) 21 (winter) 11	54.14	−2.80

^a^According to Ford et al. [46]

^b^Reflects the numbers of samples from which PCR products were successfully obtained, 22 samples were collected from each site in each season

### Molecular methods

DNA was extracted from 0.05 g of homogenised dry roots using MoBio PowerPlant DNA isolation kit following the manufacturer’s instructions (MoBio Laboratories Inc., Carlsbad, CA, USA). In order to quantify the diversity and composition of fungal communities associated with salt marsh plant roots, the internal transcribed spacer (ITS1) region was PCR-amplified from homogenised roots with the primers ITS1f and ITS2 [48, 49]. These primers target all major phyla of fungi (Ascomycota, Basidiomycota, Chytridiomycota, Glomeromycota, Mucoromycotina and Zygomycota), but exclude the unicellular animal–parasite group Microsporidia. PCR products were bead purified using Agencourt AMPure XP PCR Purification beads (Beckman Coulter Ltd, High Wycombe, UK), before sample-specific Nextera XT indices were added to amplicons with a short (8) cycle PCR. After pooling samples in equimolar concentrations, sequencing was conducted on an Illumina HiSeq 2500 in rapid run mode (providing 2 × 300 bp sequences) at The Earlham Institute (formerly The Genome Analysis Centre, Norwich Research Park, Norwich, NR4 7UH, UK). A more detailed description of molecular workflows is available in the Supplementary Information (Methods S1).

### Bioinformatic analyses

Analyses were carried out on forward reads only, as paired-end overlapping of sequences was not possible due to the length of the sequenced amplicon [50]. Sequences were quality filtered with minimum quality threshold of Q20 using Qiime [51], and clustered into (97% similarity) OTUs with VSEARCH [52], following protocols described by Dumbrell et al. [53]. Taxonomy was assigned to OTUs with the RDP classifier, set to “fungalits_unite” mode [54, 55]. Fungal OTUs were then assigned to functional (trophic) groups using FUNGuild [56]. Trophic group here refers to the nutrient acquisition strategy of the fungus, which may be pathotrophic (obtain nutrients by harming host cells), saprotrophic (obtain nutrients from dead organic matter), symbiotrophic (obtain nutrients by exchange with host) or a combination of these, reflecting multiple feeding strategies, resulting in six possible trophic groups.

Further details of bioinformatic analyses can be found in the Supplementary Information (Methods S1). Raw demultiplexed sequence data have been uploaded to the European Nucleotide Archive (accession number PRJEB20364).

### Statistical analyses

In order to examine whether abiotic or biotic factors better predict the richness and abundance of OTUs within each site, we compared statistical models (details below) using either abiotic or biotic variables (Table S1). We selected variables for each model if there was strong evidence of them being drivers of environmental fungal diversity and community structure from the literature. Abiotic models included the variables: site (when analysing data at the regional or overall scales, Fig. 1b), season [19, 26], salinity [43, 45], pH [17, 25, 57, 58] and soil moisture [34, 59, 60]. Biotic models consisted of the variables: plant species richness [25, 26, 29], total root biomass [29] and percentage cover of herbs/forbs, shrubs, grasses, sedges and rushes [28, 38, 61]. For percentage cover variables, we grouped plant species into herbs/forbs, shrubs, grasses, sedges and rushes as these groups reflect the broad differences in root morphology and life history strategies (e.g., perennial vs. annual) that we expected to affect fungal communities [25, 26, 62]. This modelling framework provides a more biologically intuitive interpretation of relationships between explanatory and response variables than analyses that rely on first reducing the dimensionality of the multiple explanatory variables (e.g., via principal components analysis (PCA)). Moreover, statistical models describing fungal diversity constructed on data from a given site/scale can be used to forecast predictions of fungal diversity at other sites/scales addressing Hypothesis H1b. This would not be possible if we first reduced the dimensionality of our explanatory variables, as ordination scores and loadings would change between sites/spatial scales. Plant classifications followed those of Rose [63, 64]. All statistical analyses were carried out in R (version 3.4.4) [65].

### Influence of the biotic and abiotic environment on fungal diversity

To test the hypothesis that drivers of fungal richness will change between sites and spatial scales, we applied a generalised linear modelling (GLM) approach to model fungal OTU richness as a function of biotic or abiotic variables. Analyses were conducted in a spatially nested manner (Fig. 1b) by modelling OTU richness within each site, then within each region (by pooling data from different sites in the same region) and then overall (by pooling all data), enabling comparisons between sites and at different spatial scales. Negative binomial GLMs were used to model OTU richness, and unequal library sizes were accounted for by including log(library size) as the first term in each model. Abiotic and biotic models were compared using Akaike’s Information Criterion (AIC) and adjusted D^2^ [66]. To test the generality of OTU richness models (H1b; that extrapolating models of fungal richness would result in poor predictions), models were parameterised (trained) on each site as described, and then applied to environmental data from the other sites to predict OTU richness. The ability of models to predict OTU richness in other sites was quantified using predictive error (root-mean-square error), between predicted and observed values. Models that generalise well, accurately predicting OTU richness in other sites, will have low predictive error. To determine whether models generalise better within regions than between regions, ANOVA tests were conducted on log transformed predictive errors.

### Influence of the biotic and abiotic environment on fungal abundances

To test our hypothesis that the relative importance of biotic and abiotic variables to fungal community structure will also differ across sites and scales, we modelled fungal community composition in each site using multivariate negative binomial GLMs [67]. We treated the number of sequences in each OTU as its abundance, and included library size as an offset term. An offset term is a model term for which the coefficient is fixed at 1, rather than estimated. This is commonly used in ecological studies to account for different sampling depths, and here, we utilise this to incorporate the effects of varying library sizes on the counts of OTUs by assuming proportionality between OTU counts and the library size of each sample [68, 69]. Whilst these numbers are not truly quantitative, by also considering overall library sizes in our models they effectively represent relative, rather than absolute, abundance. The fit of biotic and abiotic models was compared using OTU-specific AIC scores. A model was considered to have support over the other model, if the difference in AIC (ΔAIC) > 2 [70]. The total AIC across all OTUs (ΣAIC) for each model was calculated to make comparisons at the community level. These ΣAIC comparisons are only valid for comparing between models for a given site/scale, and not valid for comparing models across sites/scales as dependent variables differ. As with the OTU richness models, OTU abundance modelling was conducted in a spatially nested manner (Fig. 1b).

### Consistency of environmental responses within functional groups

To test whether functionally similar fungi show similar relationships to biotic or abiotic variables, we first grouped fungal OTUs into ecogroups. To do this, we used finite mixture modelling [71]. These models require a user-specified number of groups (originally referred to as “archetype species” [71] and “ecogroups” here for clarity) into which OTUs are clustered based on their modelled response to environmental gradients, which was set to equal the number of fungal functional groups (6). This was done to allow for a “maximal association” scenario, whereby each functional group corresponded to exactly one ecogroup. For this analysis, data from all sites were pooled (Fig. 1b) and rarefied OTU abundances (12,738 sequences per sample) were modelled independently with biotic or abiotic variables, as described above. OTUs were assigned to whichever ecogroup achieved the highest membership probability. A contingency table was calculated, summarising the number of OTUs from each functional group in each ecogroup. To test for the association between these two classifications, Chi-Squared tests were used, with *P*-values calculated on 10,000 permutations. For clarity, ecogroups were defined independently for biotic and abiotic variables, thus abiotic ecogroup 1 is independent from biotic ecogroup 1.

Further details of statistical methods used are described in Methods S2.

## Results

From the 218 salt-marsh plant-root samples from which PCR products were successfully obtained, we recovered ~99.6 million ITS DNA sequences which, after stringent quality filtering, were reduced to ~55.8 million sequences. One sample was excluded from further analyses due to insufficient sequence numbers. Remaining samples had a median of 170,349 sequences. These sequences clustered into 4638 non-singleton OTUs. Most OTUs were assigned to a phylum, whilst ~25% were identified to species level (Fig. S1). Taxonomic assignments showed that most sites were dominated by 3–4 fungal classes, with the Sordariomycetes, Dothideomycetes and Agaricomycetes particularly abundant (Fig. S2). Functional assignments of fungal OTUs revealed that the majority of OTUs were not assigned to a functional group (Fig. S3). Of those that were assigned, saprotrophs were the most abundant, followed by symbiotrophic and pathogenic fungi.

**Fig. 2:**
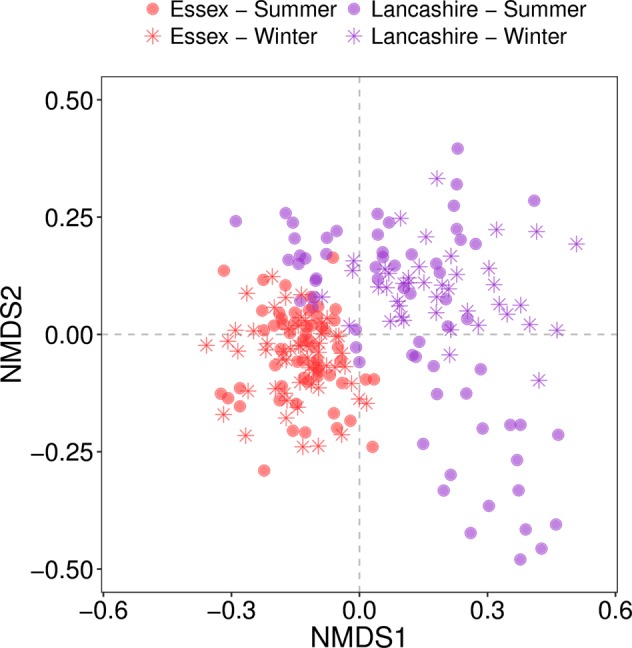
NMDS plot of similarity between fungal communities, based on Jaccard’s dissimilarity index. Each point represents a root-associated fungal assemblage collected from our soil cores. The closer points are, the more similar their community composition. Fungal communities showed clear regional distinctions, but seasonal differences were more subtle

Approximately 33% of all OTUs were shared between Essex and Lancashire sites. However, Lancashire contained more unique OTUs than Essex (2328 unique OTUs vs. 769, respectively). Compositionally, Essex and Lancashire communities were distinct (Fig. 2) and variation between the three sites in each region was higher in Lancashire than Essex. Furthermore, winter and summer samples from Lancashire appeared to contain compositionally distinct communities, whilst there was no clear separation between communities sampled in winter and summer from Essex.

**Fig. 3:**
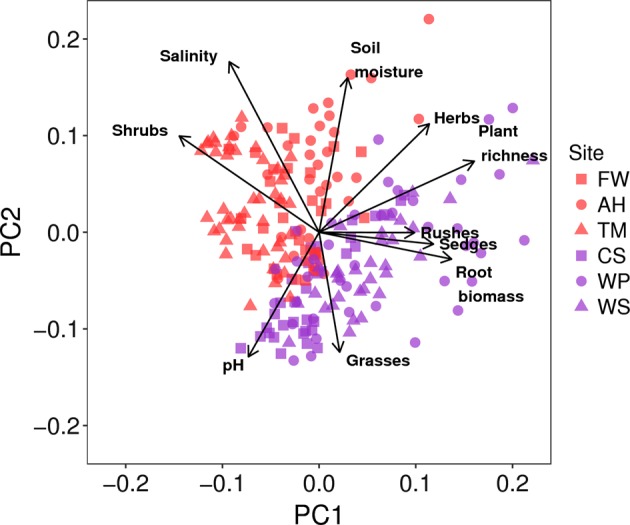
A biplot of principal component analyses (PCA) on abiotic and biotic variables across the six study sites. Essex marshes (red points) appear distinct from Lancashire marshes (purple points), whilst differences between individual sites appear to be driven by specific environmental variables

In terms of environmental variability, the six salt marshes differed in both biotic and abiotic characteristics (Fig. 3). Essex salt marsh sediments were notably more saline compared with Lancashire sediments (Table S2), but also more variable (Fig. S4), whilst a similar range of sediment pH and moisture values were observed across the regions. With respect to the plant communities, Essex marshes contained a greater abundance of shrub species, whereas Lancashire marshes were dominated by grasses, sedges and rushes (Table S2 and Fig. 3). Notably, variability in biotic variables tended to increase at larger spatial scales (e.g., regional and overall, compared with site), and more markedly than abiotic variables (Fig. S4).

### Biotic and abiotic drivers of fungal richness

**Fig. 4:**
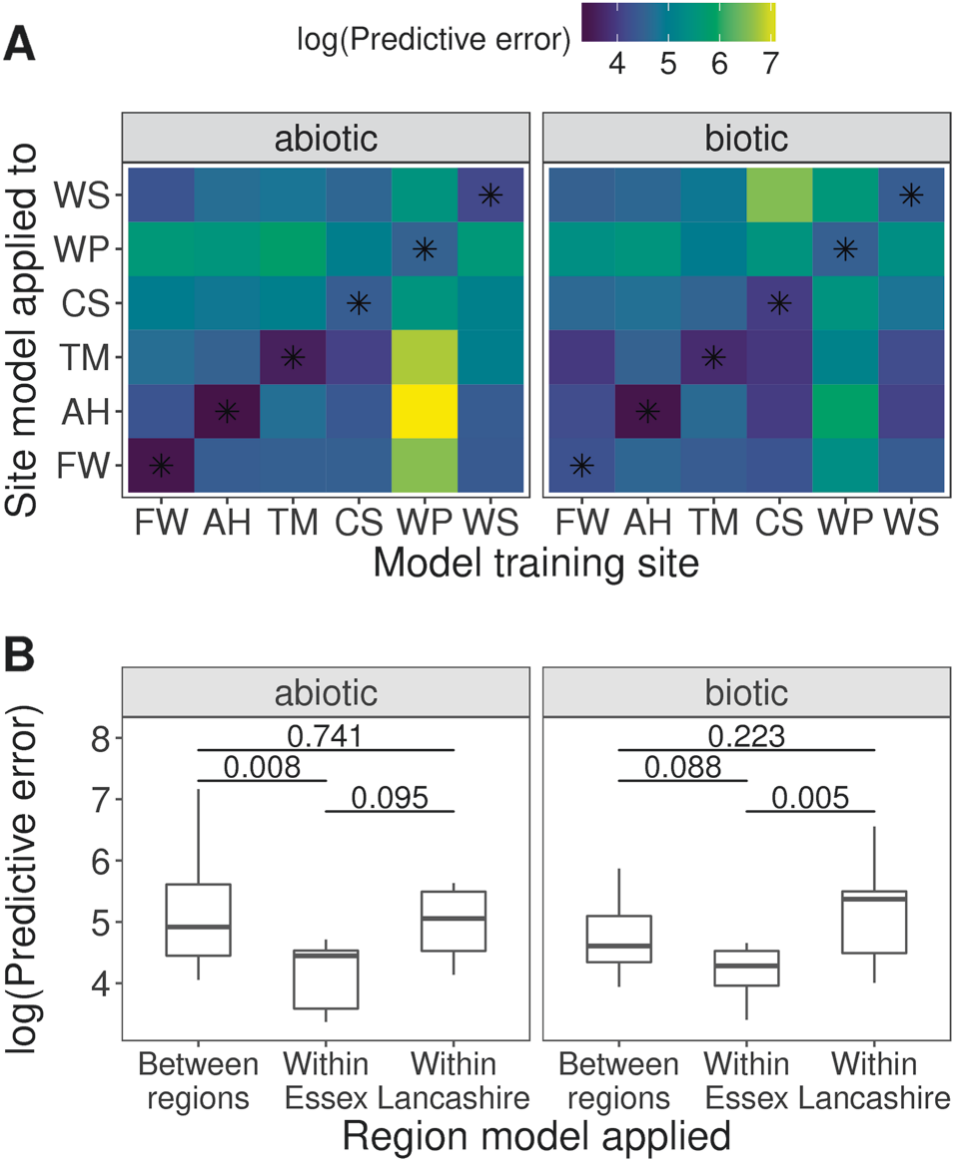
Predictive error of operational taxonomic unit (OTU) richness models when used to predict other sites’ OTU richness. **a** The predictive error of an OTU richness model when trained on data from a given site (*x*-axis) and used to predict OTU richness in another site (*y*-axis). Asterisks indicate the predictive error of models which were trained and applied on the same site. Lower values indicate closer fit between observed and predicted values. Site labels are as follows: AH   Abbotts Hall, FW   Fingringhoe Wick, TM  Tillingham Marsh, CS  Cartmel Sands, WP West Plain, WS  Warton Sands. **b** Predictive error of models when applied to a site from a different, or the same, region to the site from which they were trained. Horizontal lines and numbers represent group-wise comparisons, and the corresponding adjusted Tukey *P-*values

At the site level, abiotic variables were better predictors of OTU richness than biotic variables in all sites except Cartmel Sands. However, the difference in the fit of these models varied considerably by site (Fig. 4) according to AIC and adjusted D^2^. Furthermore, the identity and direction of relationships with individual biotic or abiotic variables was markedly different between sites (Table 2 and S3). For example, OTU richness was significantly higher in winter than summer at Fingringhoe Wick (coefficient = 0.87, *P*<0.001) and Warton Sands (coefficient = 0.83, *P<*0.001), but significantly lower at West Plain (coefficient = −0.36, *P<*0.01). Salinity and pH were only significant predictor variables in one site each (Tillingham and Cartmel Sands, respectively), and none of the abiotic variables showed statistically significant relationships in Abbotts Hall. Similarly, within the biotic variables, root biomass was correlated positively to OTU richness in Warton Sands (coefficient = 0.05, *P* = 0.05), but correlated negatively to it in West Plain (coefficient = −0.02, *P<*0.01). The environmental drivers of fungal diversity are therefore highly dependent on site at the smallest spatial scale within our study.

**Table 2:** AIC and adjusted D^2^ of fungal operational taxonomic unit (OTU) richness models loaded with abiotic and biotic variables for each site and spatial scale

Site/Scale	Abiotic variables		Biotic variables	
	AIC	Adj-D^2^	AIC	Adj-D^2^
Fingrinhoe Wick	309.4	0.82	368.4	0 (−0.14)
Abbotts Hall	353.6	0.24	358.6	0.15
Tillingham	442.9	0.54	458.2	0.36
Cartmel Sands	395.9	0.25	379.8	0.54
West Plain	466.5	0.57	467.3	0.58
Warton Sands	369.8	0.42	390	0 (−0.03)
Essex	1175.2	0.41	1211.7	0.18
Lancashire	1262.3	0.52	1288.4	0.39
Overall	2444.4	0.59	2509.9	0.44

A lower AIC and higher adjusted D^2^ indicate better model fit

Also, at a regional scale, abiotic variables were superior predictors of OTU richness than biotic variables, although the difference in performance was less marked in Lancashire compared with Essex (Table 2). Similarly to the site level, the relationships between individual variables and OTU richness were dependent on the region. In Essex, aside from site–site differences in OTU richness, no abiotic variables were significantly related to OTU richness. In contrast, beyond differences accounted for by site, salinity and soil moisture were both significantly related to OTU richness within Lancashire. No biotic variables were significantly related to OTU richness within Essex, but root biomass (coefficient = 0.02, *P*<0.05) and percentage cover by rushes (coefficient = −0.01, *P*<0.01) were both significant in Lancashire. Results at the regional scale therefore suggest that drivers of fungal diversity are similarly context-dependent at larger spatial scales.

When all data were pooled, the model loaded with abiotic variables better predicted fungal OTU richness, compared with the biotic model (Table 2). Despite this, site-specific differences were the only statistically significant abiotic relationship, and no other individual abiotic variables were significantly related to fungal OTU richness. Contrastingly, plant species richness, root biomass and percentage cover by herbs, shrubs, grasses and rushes, all showed significant relationships with fungal OTU richness.

To further test the context-dependency of drivers of fungal diversity, site level models of OTU richness were used to predict OTU richness in other sites. In support of the different drivers across sites identified previously, most models performed poorly when predicting OTU richness in other sites (Fig. 4a). Unsurprisingly, models had the least predictive error when trained and applied within the same region (Fig. 4b; ANOVA, biotic; F_2,33_ = 5.70, *P*<0.01, abiotic; F_2,33_ = 5.26, *P*<0.05). Models trained and applied to Essex sites made better predictions than those trained and applied within Lancashire, although this difference was only significant for biotic variables (biotic models; *P*<0.01, abiotic models; *P* = 0.09), suggesting that whether, or not, these drivers are generalisable may depend on the floristic or environmental similarity between sites.

### The relative roles of the biotic and abiotic environment on fungal community composition

**Fig. 5:**
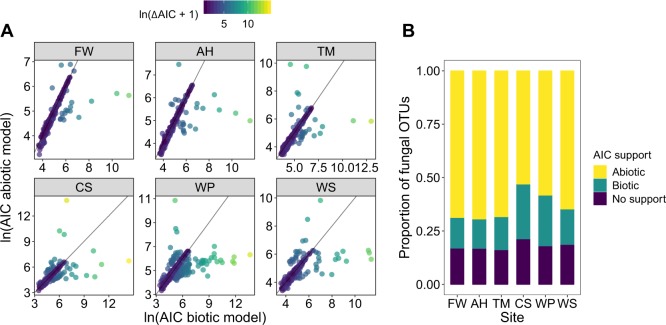
**a** The AIC scores of biotic and abiotic models for fungal operational taxonomic units (OTUs) in each salt marsh. A lower AIC score represents a superior fit (relative to the number of variables in each model). Solid lines indicate equal AIC scores for the two models. For visual clarity, OTUs with an AIC difference of <2 have been removed. **b** The proportion of fungal OTUs in each site whose abundance was better modelled with either biotic or abiotic variables. As above, “No support” indicates OTUs for which the difference in AIC between the two models was < 2, indicating that neither model was sufficiently better than the other

In partial agreement with our hypothesis (H2), the proportion of OTUs whose abundance was predicted better by biotic or abiotic variables differed between sites, although in all sites, more OTUs were predicted better by abiotic variables, than by biotic variables (Fig. 5, Table S4). In contrast, at the regional level this trend reversed, as the abundances of most OTUs were better modelled by biotic variables with 71.9% (Essex) and 66.4% (Lancashire) of OTUs having AIC support for biotic variables compared with 13.9% (Essex) and 19.4% (Lancashire) for abiotic variables. Similarly to the site level, ΣAICs for each model in each region still supported abiotic variables in both Essex and Lancashire (Table S4). This shows that for OTUs whose abundance was predicted better by abiotic OTUs, the difference in AIC outweighed the majority of OTUs, whose abundance was predicted better by biotic variables. At the largest scale (overall) in our study, abiotic variables were again better predictors of OTU abundances as 1164 of the 1999 OTUs analysed showed support for abiotic variables, compared with 588 for biotic variables (Table S4). At this spatial scale, ΣAIC for each set of variables also supported abiotic variables (ΣAIC = 4,353,897) over abiotic variables (ΣAIC = 14,674,391).

### Environmental responses of functional groups

**Fig. 6:**
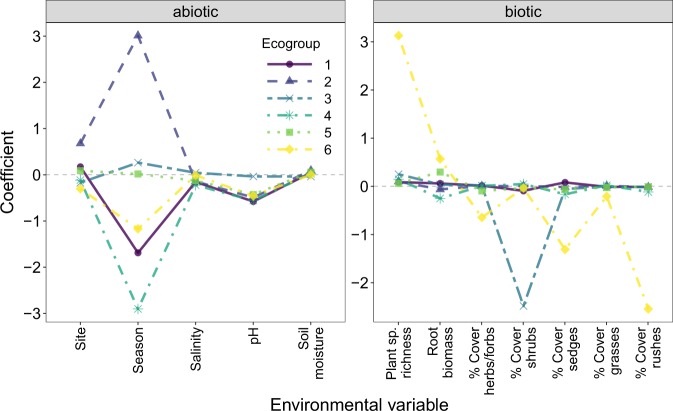
The estimated coefficients of fungal ecogroups in relation to biotic and abiotic environmental variables. Coefficient values of 0 indicate little relationship between the given environmental variable and the abundance of fungal operational taxonomic units (OTUs) within each ecogroup. Ecogroups were created independently for biotic and abiotic variables

**Fig. 7:**
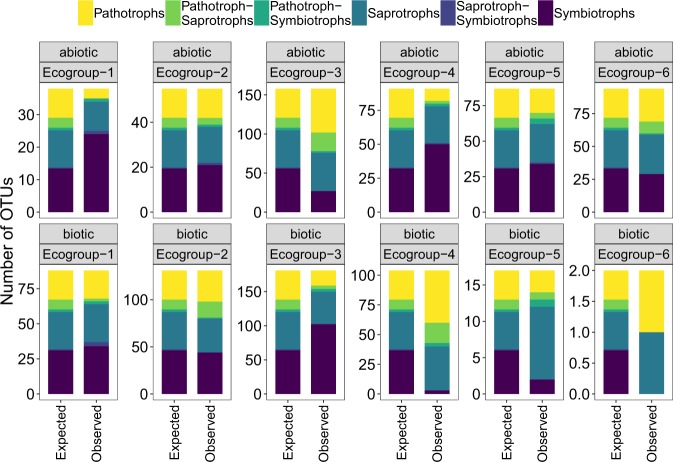
The expected and observed number of fungal operational taxonomic units (OTUs) from each functional group in each ecogroup. Ecogroups were created from finite-mixture models with either abiotic (upper panels) or biotic variables (lower panels) independently. Expected numbers are calculated under the null hypothesis of no association between functional groups and ecogroups

Finite-mixture models were used to group OTUs into ecogroups based on their modelled response to environmental variables. These ecogroups showed markedly different ecological preferences according to their modelled responses to biotic or abiotic variables (Fig. 6). Biotic ecogroups were differentiated by their predicted relationships with root biomass, whilst other differences between ecogroups were more specific to certain biotic variables. For example, biotic ecogroup 1 showed a notably stronger positive relationship with plant species richness, whereas biotic ecogroup 3 showed a far stronger negative relationship to shrub cover than other ecogroups (Fig. 6). Abiotic ecogroups showed considerably different seasonal dynamics (e.g., ecogroups 2 and 4), whereas differences between the ecogroups in response to other abiotic variables were more subtle. Abiotic ecogroup 3 showed little response to pH, whereas most other abiotic ecogroups had negative relationships between their abundance and pH (Fig. 6). The extent by which the functional composition of ecogroups differed from expected was variable (e.g., abiotic ecogroup 1 vs. 2, Fig. 7). However, for both (biotic and abiotic) sets of ecogroups, Fisher’s exact tests revealed significant association between fungal functional groups and ecogroups (biotic; Fisher’s *P<*0.001, abiotic; Fisher’s *P*<0.001). This indicates that ecogroups are made up of disproportionate numbers of OTUs from each functional group. Consequently, a change to any of these variables would be predicted to disproportionately affect the abundance of certain functional groups.

## Discussion

### Salt marsh fungal communities

This study significantly extends our knowledge of the diversity and ecology of root-associated fungi in the natural environment. We analysed > 55 million fungal ITS gene sequences from 218 samples from six different UK salt marshes, and found a highly diverse mycobiome comprising > 4000 OTUs. This represents a fourfold increase in the diversity of fungi previously recorded in salt marsh habitats [44], and is comparable with forests [58, 72] and tropical soils [73], implicating salt marshes as significant repositories of fungal biodiversity. Furthermore, our study is one of a few that have attempted to understand the drivers of root-associated fungal diversity in salt-marsh environments. Our analyses reveal complex relationships between the environment and fungal community structure and diversity, with few, if any, general unifying relationships, suggesting that context-dependency is an important aspect of fungal ecology that deserves greater attention.

### Abiotic variables determine fungal richness, but not in a generalisable manner

Across sites and spatial scales, fungal OTU richness was more closely linked to the abiotic, than the biotic, variables. However, the fit, direction of relationships, and statistical significance of these relationships were highly context-dependant, changing between sites and scales. In contrast to previous research, we found relatively few statistically significant relationships between abiotic variables and fungal diversity. Salt marshes present gradients of salinity in relation to tidal exposure and successional stage, that may influence fungal diversity [43, 44, 74, 75]. Yet, we observed an inconsistent relationship between salinity and fungal diversity that varied between regions, with negative relationships in Lancashire, and no relationship in Essex sites. Notably, Lancashire sites were less saline and with less variability in salinity than Essex sites (Table S2; Fig. S4) and therefore, the regional fungal metacommunity may contain fewer halo-tolerant species.

Differences in OTU richness between winter and summer samples were observed in multiple sites, but the magnitude and direction of these differences were site dependent, reflecting similar inconsistencies observed across studies [19, 26, 76]. The fungi recorded here comprise various trophic modes (symbiotrophic, saprotrophic and pathotrophic), which target different plant-derived resources. Thus, seasonal patterns in the availability of live and dead biomass (e.g., ref. [77]) could drive shifts in diversity by stimulating different fungal trophic pathways. As seasonal dynamics of living and dead plant biomass are species-specific [78], differences in floristic composition across sites, could potentially explain site-specific changes in fungal diversity between winter and summer samples.

Few biotic variables appeared to influence fungal richness; root biomass was only a statistically significant driver in two sites, where it had inconsistent effects on fungal richness. This was contrary to our expectation, as increased root biomass should provide greater colonisation area, thus supporting more species via a species–area relationship. Furthermore, plant species richness was only significant for the pooled Lancashire, and overall datasets, but not within any individual site. Increasing plant richness might increase fungal richness by diversifying the pool of resources and ecological niches available to fungi, although we did not observe this relationship in most sites and scales. One possibility is that root biomass and plant richness are poor proxies for the ecological niche space available to fungi. Instead, specific root traits, such as root exudates and differentiation of root morphologies, may be better predictors of root-associated fungal diversity [24, 79, 80], but are also more challenging to quantify. For example, Essex salt marshes contained a greater abundance of shrubs, which often have woody root systems that resist colonisation by arbuscular mycorrhizas (Glomeromycota) [42], thereby reducing the available niche space to AM fungi and, by extension, the number of AM fungal species.

One potential explanation for the strong degree of context-dependency observed in this study is that site- and/or scale-dependent variability in environmental parameters may determine what is perceived as most important in structuring root-associated fungal communities. For example, in sites with a greater variance in salinity, salinity may emerge as a more “statistically significant” predictor than in sites where variance in salinity is smaller, and the same would hold for other abiotic or biotic variables. This is because within the statistical framework employed here, increased variability in predictor variables would lead to greater precision of parameter estimates, and an associated decrease in P values. However, P values alone are a poor indicator of a variable’s predictive usefulness. If the form (shape and direction) of the relationships between salinity (as an example) and fungal community structure is consistent across sites, then parameter estimates would be broadly similar in each site regardless of within-site variance in salinity. Thus, while P values may vary, the form of modelled relationships and predictive usefulness of highlighted variables should not. Given the variability in estimated parameters, statistical significance and predictive performance observed within our study (Fig. 4 and Table S3), we don’t believe that observed patterns of context-dependency are merely statistical artefacts resulting from site- or scale-dependent variance in the predictor variables. Moreover, as difference across sites and scales in within-site variability of abiotic and/or biotic variables reflects local patterns of environmental heterogeneity, it forms a biologically relevant aspect of context that should be considered.

### Drivers of fungal community composition are context-specific

The relative importance of biotic and abiotic variables on fungal community composition was highly context-dependent, shifting between sites and spatial scales. Within sites, the relative abundance of most fungal OTUs was best predicted by abiotic factors, supporting previous work on the role of abiotic properties in modulating fungal community structure [17, 57, 59]. In Essex sites, the proportion of OTUs whose abundance was better predicted by abiotic factors was remarkably consistent (Fig. 5), perhaps reflecting the more similar fungal community composition between sites, as compared with Lancashire sites. This result agrees with our finding that the drivers of fungal diversity were more similar and generalisable between Essex sites, suggesting similar ecological processes in these marshes.

While abiotic factors were more important than biotic factors within sites, at the regional level this trend reversed, and biotic factors became better predictors. This is likely due to the distinct plant communities in each study region outweighing differences in abiotic variables [47]. This explanation is supported by the fact that biotic variables tended to become increasingly variable as we increased the spatial scale at which data were modelled (Fig. S4). Sediment characteristics and climate also differ notably between the two regions, but these variables are likely to be reasonably homogeneous within each site in a region. Therefore, whilst these variables could influence fungal community composition at the landscape scale [27], we suggest that plant community differences are still likely to hold more explanatory power.

As with all environmental microbial ecology studies, it is impossible to measure all of the relevant environmental parameters and, inevitably, some environmental variables may remain unmeasured. However, the primary aim of our study was not to identify the main drivers of fungal community structure per se, but rather to test their consistency between different sites and spatial scales, in which case our consistent sampling design and measurements of environmental variables are sufficient. Another potential caveat of our study is that, to some extent, differences in plant community composition are themselves related to abiotic environmental gradients, and thus, biotic effects may actually be indirect abiotic influences [81, 82]. To disentangle the effects of plant community structure from the abiotic environment would require experimental approaches, beyond the scope of this study. Instead, given that biotic variables were not strongly collinear with abiotic variables (Fig. 3), we assume that indirect effects of the abiotic environment acting on plant community structure are reflected in the abiotic variables themselves, and that relationships between biotic variables and fungal communities are not confounded.

Our analyses of the relationships between fungal diversity and community structure and the environment show high levels of context-dependency. Consequently, extrapolating models from one site to another resulted in poor quality predictions of fungal diversity. Previous research has hinted that drivers of microbial diversity are dependent on both environmental and ecological context, with few generalisable predictors [25, 83]. However, this study is the first to explicitly test this. Our results show that context-dependency may hinder the search for unifying “macro-ecological” relationships in microbial ecology and that seeking to understand drivers of community structure from a single site or spatial scale is unwise. Despite this, we also found aspects of fungal community ecology that do appear to generalise across ecological and environmental contexts. For example, within most sites, abiotic variables were more important predictors of fungal diversity and community structure than biotic variables. Thus, measuring relevant aspects of the abiotic environment should be prioritised if attempting to predict fungal diversity or community structure within a site.

### Functional group is an important contextual aspect of fungal ecology

We observed an association between fungal ecogroups and functional groups. Therefore, how environmental gradients influence fungal taxa depends on their function, as is commonly observed in experimental studies that show differential responses between fungal functional groups to warming [84, 85], nitrogen addition [86], CO_2_ [87] and plant species richness and identity [26, 38, 88].

Whilst substantial experimental evidence exists for differing ecologies across fungal functional groups, few studies have demonstrated this in natural settings. The few observational studies that explicitly examine the ecologies of functionally dissimilar groups of microorganisms, tend to rely on broad taxonomy as a proxy for function. For example, Powell et al. [89] observed varying roles for niche and neutral community assembly mechanisms between bacteria and fungi in soils, with fungal communities less predictable by niche processes and more prone to stochastic neutral assembly. However, in these studies taxonomy and functionality may be confounding each other at such a broad taxonomic resolution. A more specific study was conducted by Peay et al. [73], who found contrasting responses of fungal functional groups to plant species richness. Specifically, they observed that the richness of fungal groups whose trophic mode primarily depended on the host plant (mycorrhizal and pathogenic fungi) showed positive relationships with plant diversity, whereas saprotrophic fungi (which do not depend primarily on plants) were largely invariant to plant diversity. Similarly, Mommer et al. [90] showed contrasting responses of plant pathogenic and endophytic fungi to increased levels of plant species richness. These results support our finding that functionally distinct fungi show differing ecological preferences in response to single or multiple environmental gradients. Consequently, moving from taxonomy-, to functional trait-based approaches may provide a more generalisable framework for understanding fungal community ecology, as is the case in “macro-organismal” research [91].

In salt-marsh ecosystems, the potential for environmental change to differentially influence the relative abundance of different fungal functional groups could have major implications for ecosystem processes and functions. For example, abiotic ecogroup 1 was composed of ~1.5 times the expected number of symbiotrophic fungal OTUs. This ecogroup was found to have a negative predicted relationship with pH, suggesting that fungi in this group will generally increase in abundance in more acidic sediments. Whilst the impacts of climate change on salt-marsh sediments are largely focussed on carbon cycling, acidification of coastal waters coupled with increased inundation as sea levels rise may decrease sediment pH. In turn, this may increase the abundance of certain symbiotrophic fungi, and by extension may up-regulate the ecosystem processes they contribute to (e.g., ref. [92]). However, more in-depth studies are still required to determine the functionality of many plant-associated fungal groups [93], and the relationship between their abundance and ecosystem processes [94].

## Conclusions

Our study of fungal communities from six UK salt marshes revealed highly context-dependent drivers of fungal community structure and diversity. By carrying out a spatially replicated study, we found that abiotic variables were generally superior predictors of community structure and diversity across sites and spatial scales. Yet, the identity and direction of relationships differed between sites and spatial scales to such an extent that extrapolating them to other sites generally resulted in poor predictions of fungal diversity. Furthermore, we detected associations between fungal responses to abiotic and biotic variables and the functional groups these fungi belong to. This may suggest that environmental gradients have the potential to effect fungal functional groups differentially. Combined, our results highlight that site, spatial scale and functional group are important contextual aspects that alter the community ecology of fungi. Therefore, understanding which aspects of fungal community ecology can be generalised across sites, spatial scales or functional groups is critical for managing ecosystems and the process they support in the face of environmental change. Appropriate replication with respect to these contextual factors is essential to elucidate generalisable aspects of fungal ecology, and to move towards a more predictive framework of their community ecology.

## Supplementary information

Supplementry online material
