# Supplementary material for: Are drivers of root-associated fungal community structure context specific?
Source: ISME J. 2019 Jan 28;13(5):1330–44. doi: 10.1038/s41396-019-0350-y (PMC6474305; doi:10.1038/s41396-019-0350-y)
Supplement: Supplementary file 1 — Supplementry online material [file 41396_2019_350_MOESM1_ESM.docx]

**Supplementary Material**

**Title:** Are Drivers of Root-Associated Fungal Community Structure Context Specific?

A. Khuzaim Alzarhani^1^*, Dave R. Clark^1^*, Graham J. C. Underwood^1^, Hilary Ford^2^, T. E. Anne Cotton^1^† and Alex J. Dumbrell^1^

^1^School of Biological Sciences, University of Essex, Wivenhoe Park, Colchester, Essex, CO4 3SQ, UK

^2^School of Environment, Natural Resources and Geography, Thoday buildings, Bangor University, Bangor, LL57 2DG, UK

†Current address: Department of Animal and Plant Sciences, The University of Sheffield, Alfred Denny Building, Sheffield, South Yorkshire, S10 2TN, UK

* These authors contributed equally to this work.

Methods S1 – Detailed description of molecular methods and bioinformatic analyses

*Molecular methods*

DNA was extracted from 0.05 g of homogenised dry roots using MoBio PowerPlant DNA isolation kit following the manufacturer’s instructions (MoBio Laboratories Inc., Carlsbad, CA, USA). To quantify the fungal communities associated with salt-marsh plant roots, we used Illumina 2 x 300bp HiSeq (2500 in rapid run mode) amplicon sequencing of the internal transcribed spacer (ITS) region. This region is the standard amplicon of choice for molecular studies of fungal communities (e.g. Buée *et al*., 2009; Pellissier *et al*., 2014; Vannette *et al*., 2016) as it is easier to amplify than other marker genes and has good species level discrimination (Schoch *et al*., 2012). The ITS region was amplified using the primers ITS1f and ITS2 (White *et al*., 1990; Gardes and Bruns, 1993) modified to contain Illumina-specific sequencing adaptors, in 25 μl reactions using 12.5 μl REDTaq® ReadyMixTM (Sigma-Aldrich Co.), 5 μl of each (1 μM) primer and 2.5 μl of DNA template. Where necessary, 0.05 μl of T4 Gene 32 protein (Roche Diagnostics lid, W. Sussex, UK) was added to PCR reactions to prevent inhibition from humic acids or other inhibitors (Kreader, 1996). Thermal cycling consisted of an initial DNA denaturation step of 3 min at 95°C followed by 32 cycles each of 30 s at 94°C, 40 s at 58°C and 45 s at 72°C with a final extension step of 10 min at 72°C, on an Applied Biosystems Veriti 96-well thermal cycler. The resulting PCR products were purified using Agencourt AMPure XP PCR Purification beads (Beckman Coulter Ltd, High Wycombe, UK), following the manufacturer’s instructions. 5 μl of purified PCR product was used in a short cycle secondary PCR, to attach Nextera XT indices, in the presence of 5 μl of Nextera i5 and i7 index, 25 μl of REDTaq® ReadyMix™ (Sigma-Aldrich Co.) and 10 µl of PCR water (Bioline Reagents Ltd, UK). Thermal cycling conditions consisted of an initial denaturation step of 3 min at 95°C followed by 8 cycles each of 30s at 95°C, 30 s at 55°C and 30 s at 72°C followed by a final extension step of 5 min at 72°C. PCR products were purified using Agencourt AMPure XP PCR Purification beads as described previously. PCR products were quantified using PicoGreen® dsDNA quantification assays (Thermo Fisher Scientific Inc. USA), on a POLAR star Omega (BMG LABTECH GmbH, Germany) plate reader. Nextera XT amplicons were then pooled in equimolar concentration and the concentration and length of the pooled amplicon was verified on an Agilent 2100 Bio-analyser. Sequencing was conducted on an Illumina HiSeq 2500 in rapid run mode (providing 2 x 300bp sequences) at The Earlham Institute (formerly The Genome Analysis Centre, Norwich Research Park, Norwich, NR4 7UH, UK).

*Bioinformatic analyses*

Following protocols described by Dumbrell *et al*. (2016), sequences were quality filtered with minimum quality threshold of Q20, using Qiime (Caporaso *et al*., 2010). High quality, full length sequences were then stripped of the forward primer using Linux shell commands. VSEARCH version 2.1.1 (Rognes *et al*., 2016) was used to de-replicate sequences, remove singleton sequences and cluster sequences at a 97% similarity threshold. OTUs were chimera checked with VSEARCH against the 97% representative sequence dataset from the Unite database (Kõljalg *et al*., 2013). VSEARCH is an open source implementation of the popular USEARCH pipeline (Edgar, 2010) and produces comparable, high quality results (Westcott and Schloss, 2015; Jackson *et al.*, 2016). Taxonomy was assigned to OTUs using the RDP Naïve Bayesian Classifier (Wang *et al.*, 2007) trained on the UNITE database, with a minimum confidence threshold of 0.7. In order to test our third hypothesis, fungal OTUs were assigned to functional groups using FUNGuild (Nguyen *et al.*, 2016a). FUNGuild categorises fungal OTUs into trophic groups by matching taxonomic terms to a database of fungal functional traits. Trophic group here refers to the nutrient acquisition strategy of the fungus, which may be pathotrophic (obtain nutrients by harming host cells), saprotrophic (obtain nutrients from dead organic matter), symbiotrophic (obtain nutrients by exchange with host), or a combination of these, reflecting multiple feeding strategies. Here, we use trophic modes as our functional groupings as we were primarily interested in a functional categorisation that reflects groups of fungi with broadly similar effects on, or contributions to, plant communities and ecosystem functions, as such groups have been shown to have differing ecologies (Tedersoo *et al.*, 2014). Raw demultiplexed sequence data have been uploaded to the European Bioinformatic Institute database (accession number PRJEB20364).

References

Buée M, Reich M, Murat C, Morin E, Nilsson RH, Uroz S, *et al.* (2009). 454 Pyrosequencing analyses of forest soils reveal an unexpectedly high fungal diversity. *New Phytol* **184**:449–456.

Caporaso JG, Kuczynski J, Stombaugh J, Bittinger K, Bushman FD, Costello EK, *et al.* (2010). QIIME allows analysis of high-throughput community sequencing data. *Nat Methods* **7**:335–336.

Dumbrell AJ, Ferguson RMW, Clark DR. (2016). Microbial Community Analysis by Single-Amplicon High-Throughput Next Generation Sequencing: Data Analysis -- From Raw Output to Ecology. In: McGenity TJ, Timmis KN, Nogales B (eds). *Hydrocarbon and Lipid Microbiology Protocols: Microbial Quantitation, Community Profiling and Array Approaches*. Springer: Heidelberg, Germany, pp 155–206.

Edgar RC. (2010). Search and clustering orders of magnitude faster than BLAST. *Bioinformatics* **26**:2460–2461.

Gardes M, Bruns TD. (1993). ITS primers with enhanced specificity for basidiomycetes - application to the identification of mycorrhizae and rusts. *Mol Ecol* **2**:113–118.

Jackson MA, Bell JT, Spector TD, Steves CJ. (2016). A heritability-based comparison of methods used to cluster 16S rRNA gene sequences into operational taxonomic units. *PeerJ* **4**:e2341.

Kõljalg U, Nilsson RH, Abarenkov K, Tedersoo L, Taylor AFS, Bahram M, *et al.* (2013). Towards a unified paradigm for sequence-based identification of fungi. *Mol Ecol* **22**:5271–5277.

Kreader CA. (1996). Relief of amplification inhibition in PCR with bovine serum albumin or T4 gene 32 protein. *Appl Environ Microbiol* **62**:1102–1106.

Nguyen NH, Song Z, Bates ST, Branco S, Tedersoo L, Menke J, *et al.* (2016a). FUNGuild: An open annotation tool for parsing fungal community datasets by ecological guild. *Fungal Ecol* **20**:241–248.

Pellissier L, Niculita-Hirzel H, Dubuis A, Pagni M, Guex N, Ndiribe C, *et al.* (2014). Soil fungal communities of grasslands are environmentally structured at a regional scale in the Alps. *Mol Ecol* **23**:4274–4290.

Rognes T, Flouri T, Nichols B, Quince C, Mahé F. (2016). VSEARCH: a versatile open source tool for metagenomics. *PeerJ Prepr* **4**:e2409v1.

Schoch CL, Seifert KA, Huhndorf S, Robert V, Spouge JL, Levesque CA, *et al.* (2012). Nuclear ribosomal internal transcribed spacer (ITS) region as a universal DNA barcode marker for Fungi. *Proc Natl Acad Sci USA* **109**:6241–6246.

Vannette RL, Leopold DR, Fukami T. (2016). Forest area and connectivity influence root-associated fungal communities in a fragmented landscape. *Ecology* **97**:2374–2383.

Wang Q, Garrity GM, Tiedje JM, Cole JR. (2007). Naiive Bayesian classifier for rapid assignment of rRNA sequences into the new bacterial taxonomy. *Appl Environ Microbiol* **73**:5261–5267.

Westcott SL, Schloss PD. (2015). De novo clustering methods outperform reference-based methods for assigning 16S rRNA gene sequences to operational taxonomic units. *PeerJ Prepr* **3**:e1487.

White TJ, Bruns S, Lee S, Taylor J. (1990). Amplification and direct sequencing of fungal ribosomal RNA genes for phylogenetics. *PCR Protoc A Guid to Methods Appl* **18**:315–322.

Methods S2 – Description of statistical analyses

*Fungal richness models*

Negative binomial GLMs were used to account for data overdispersion (Ver Hoef and Boveng, 2007; Warton *et al.*, 2016), a property whereby the variance exceeds the mean. Since OTU richness is strongly affected by sequencing depth, it is necessary to account for variation in OTU richness caused by random heterogeneity in sequencing depth. Therefore, we accounted for unequal sequencing depth by including the log of the number of sequences in each sample as the first term in our models, a strategy that has been successfully applied in other microbial ecology studies (e.g. Bálint *et al*., 2015). Akaike's information criterion (AIC) was used to determine whether biotic or abiotic models best predicted OTU richness. Adjusted D^2^ (Guisan and Zimmermann, 2000) was also calculated for each model. Unlike AIC, adjusted D^2^ allows comparisons, not only between different models on the same dataset, but also between models on different datasets. Negative adjusted D^2^ values were adjusted to 0 to aid interpretation. To test the generality of OTU richness models (H1b), models were parameterised (trained) on each site as described, and then applied to environmental data from the other sites to predict OTU richness. The ability of models to predict OTU richness in other sites was quantified using predictive error (root mean square error), between predicted and observed values. Models that generalise well, accurately predicting OTU richness in other sites will have low predictive error. To determine whether models generalise better within regions than between regions, ANOVA tests were conducted on log transformed predictive errors.

*Fungal community models*

To model fungal OTU abundances, the number of sequences for each OTU was treated as its abundance and OTUs with fewer than five occurrences were excluded as these OTUs are unlikely to provide enough information to construct robust models. Here, unequal sequencing depth was accounted for by including the log of the number of sequences as an offset, rather than as a free parameter (Zuur *et al.*, 2009). Use of an offset term implies proportionality between sequencing depth and the dependent variable, which is realistic for OTU abundances, but not for OTU richness (i.e. doubling the sequence depth may not double the OTU richness, but would double an OTU’s abundance). The fit of biotic and abiotic models was compared using OTU specific AIC scores. A model was considered to have support over the other model, if the difference in AIC (ΔAIC) > 2 (Burnham and Anderson, 2002). The total AIC across all OTUs (ΣAIC) for each model was calculated to make comparisons at the community level. As with the OTU richness models, OTU abundance modelling was conducted in a spatially nested manner.

*Fungal functional models*

These models are an extension of multivariate GLMs, which in our case, allowed us to cluster OTUs into ecogroups by their modelled response to environmental covariates. This method of grouping species by their environmental response has several advantages. Firstly, this method improves the predictive ability of models for low abundance species (Hui *et al.*, 2013). Secondly, it allows the grouping of OTUs based on their response to multiple environmental gradients simultaneously, which is a realistic representation of the highly dimensional niche space in which they exist (Hutchinson, 1957). Finally, this model-based approach yields useful and biologically relevant extra information such as the regression coefficients for each of the ecogroups, allowing us to understand how these groups respond to the environment, and ecogroup membership probabilities for each OTU, allowing us to quantify the confidence with which we assign fungal OTUs to ecogroups (Hui *et al.*, 2013).

These models require a user-specified number of groups into which OTUs are clustered (referred to as “archetype species” in Dunstan *et al*., (2013) and ecogroups here for clarity), which was set to equal the number of fungal trophic groups (6). This was done to allow for a “maximal association” scenario whereby each trophic group corresponded to exactly one environmental response group. For this analysis, data from all sites were pooled and OTU abundances were modelled independently with biotic or abiotic variables, as described previously. OTUs were assigned to the ERG for which membership probability was maximised. A contingency table was calculated, summarising the number of OTUs in each of the ecogroups and functional groups. To test for association between these two classifications, Fisher’s exact tests were used, with *P* values calculated on 10,000 permutations.

References

Bálint M, Bartha L, O’Hara RB, Olson MS, Otte J, Pfenninger M, *et al.* (2015). Relocation, high-latitude warming and host genetic identity shape the foliar fungal microbiome of poplars. *Mol Ecol* **24**:235–248.

Burnham KP, Anderson DR. (2002). Model Selection and Multimodel Inference: A Practical Information-Theoretic Approach. 2nd Editio. Springer-Verlag: Berlin.

Dunstan PK, Foster SD, Hui FKC, Warton DI. (2013). Finite Mixture of Regression Modeling for High-Dimensional Count and Biomass Data in Ecology. *J Agric Biol Environ Stat* **18**:357–375.

Guisan A, Zimmermann NE. (2000). Predictive habitat distribution models in ecology. *Ecol Modell* **135**:147–186.

Hui FKC, Warton DI, Foster SD, Dunstan PK. (2013). To mix or not to mix: Comparing the predictive performance of mixture models vs. separate species distribution models. *Ecology* **94**:1913–1919.

Hutchinson GE. (1957). Concluding Remarks. In: Vol. XXII. *Cold Spring Harbor Symposia*. pp 415–427.

McMurdie PJ, Holmes S. (2014). Waste not, want not: why rarefying microbiome data is inadmissible. *PLoS Comput Biol* **10**:e1003531.

Ver Hoef JM, Boveng PL. (2007). Quasi-poisson vs. negative binomial regression: How should we model overdispersed count data? *Ecology* **88**:2766–2772.

Warton DI, Lyons M, Stoklosa J, Ives AR, Schielzeth H. (2016). Three points to consider when choosing a LM or GLM test for count data. *Methods Ecol Evol* **7**:882–890.

Zuur AF, Ieno EN, Walker NJ, Saveliev AA, Smith GM. (2009). Mixed effects models and extensions in ecology with R. Springer: New York, USA.

Table S1. Details of abiotic and biotic model formulae at different spatial scales and different response variables.

| Response variable/  spatial scale | OTU richness | OTU abundances |
| --- | --- | --- |
| Site | log(library size) + season + salinity + pH + soil moisture  log(library size) + plant richness + root biomass + herbs/forbs + shrubs + sedges + grasses + rushes | season + site + salinity + pH + soil moisture + offset(log(library size))  plant richness + root biomass + herbs/forbs + shrubs + sedges + grasses + rushes + offset(log(library size)) |
| Regional | log(library size) + season + site + salinity + pH + soil moisture  log(library size) + plant richness + root biomass + herbs/forbs + shrubs + sedges + grasses + rushes | season + site + salinity + pH + soil moisture + offset(log(library size))  plant richness + root biomass + herbs/forbs + shrubs + sedges + grasses + rushes + offset(log(library size)) |
| Overall | log(library size) + season + site + salinity + pH + soil moisture  log(library size) + plant richness + root biomass + herbs/forbs + shrubs + sedges + grasses + rushes | season + site + salinity + pH + soil moisture + offset(log(library size))  plant richness + root biomass + herbs/forbs + shrubs + sedges + grasses + rushes + offset(log(library size)) |

Table S2. Biotic and abiotic characteristics of each site. Presented values are means ± one standard error. For plant species richness, the median and range (min, max) are presented.

| Model | Variable | Essex | | | Lancashire | | |
| --- | --- | --- | --- | --- | --- | --- | --- |
|  |  | Fingringhoe Wick | Abbotts Hall | Tillingham | Cartmel Sands | West Plain | Warton Sands |
| Abiotic | Salinity (ppt) | 31.2 ± 2.5 | 43.2 ± 3.2 | 33.8 ± 2.2 | 5.9 ± 0.4 | 3.3 ± 0.3 | 4.4 ± 0.4 |
|  | pH | 7.2 ± 0.1 | 7.1 ± 0.1 | 7.6 ± 0.0 | 8.0 ± 0.1 | 7.0 ± 0.1 | 7.6 ± 0.1 |
|  | Soil moisture (%) | 36.3 ± 3.6 | 40.9 ± 3.3 | 29.8 ± 2.6 | 34.5 ± 1.3 | 34.9 ± 2.4 | 26.9 ± 1.7 |
| Biotic | Plant species richness | 4 (3, 6) | 5 (2, 7) | 4 (2, 6) | 3 (1, 7) | 5 (1, 13) | 5 (2, 7) |
|  | Root biomass (g per top 30 cm soil) | 3.0 ± 0.5 | 3.5 ± 0.4 | 1.4 ± 0.2 | 2.3 ± 0.4 | 8.9 ± 0.9 | 5.2 ± 0.6 |
|  | % cover – herbs and forbs | 16.3 ± 2.4 | 26.5 ± 4.1 | 7.2 ± 0.9 | 13.2 ± 2.7 | 13.8 ± 2.5 | 22.6 ± 3.3 |
|  | % cover - shrubs | 29.3 ± 3.8 | 16.9 ± 2.4 | 58.4 ± 4.4 | 0 ± 0 | 0 ± 0 | 0 ± 0 |
|  | % cover - sedges | 0 ± 0 | 0 ± 0 | 0 ± 0 | 0 ± 0 | 2.4 ± 0.7 | 0.8 ± 0.6 |
|  | % cover - grasses | 58.0 ± 3.6 | 56.4 ± 4.3 | 29.4 ± 4.1 | 69.1 ± 3.4 | 49.9 ± 5.3 | 48.3 ± 4.9 |
|  | % cover rushes | 0 ± 0 | 0 ± 0 | 0 ± 0 | 2.1 ± 1.1 | 3.3 ± 1.4 | 17.1 ± 3.8 |

Table S3. Details of GLMs relating OTU richness to abiotic, or biotic variables. For marginally significant *P*-values (0.05 < *P* < 0.1), the actual value is presented.

| Site/  Scale | Model | Significant predictors | Coefficient | Wald  z-value | *P-*value |
| --- | --- | --- | --- | --- | --- |
| FW | abiotic | log(no. seqs)  season (Winter) | 0.12  0.87 | 3.30  4.16 | < 0.001  < 0.0001 |
|  | biotic | log(no. seqs) | 0.17 | 1.98 | < 0.05 |
| AH | abiotic | log(no. seqs) | 0.17 | 3.33 | < 0.001 |
|  | biotic | log(no. seqs) | 0.21 | 3.93 | < 0.0001 |
| TM | abiotic | log(no. seqs)  salinity | 0.19  0.02 | 4.88  3.26 | < 0.0001  < 0.01 |
|  | biotic | log(no. seqs) | 0.25 | 5.54 | < 0.0001 |
| CS | abiotic | log(no. seqs)  pH | 0.21  -0.17 | 3.03  -1.9 | < 0.01  0.055 |
|  | biotic | log(no. seqs)  % cover – rushes | 0.16  0.04 | 2.95  4.07 | < 0.0001  < 0.0001 |
| WP | abiotic | log(no. seqs)  season (Winter) | 0.26  -0.36 | 5.94  -2.75 | < 0.0001  < 0.01 |
|  | biotic | log(no. seqs)  root biomass  % cover – herbs/forbs | 0.19  -0.02  0.01 | 3.92  -2.94  3.40 | < 0.0001  < 0.01  < 0.001 |
| WS | abiotic | season (Winter) | 0.83 | 5.51 | < 0.0001 |
|  | biotic | root biomass | 0.05 | 1.92 | 0.054 |
| Essex | abiotic | log(no. seqs)  site (FW)  site (TM) | 0.18  0.35  0.34 | 5.24  4.79  4.02 | < 0.0001  < 0.0001  < 0.0001 |
|  | biotic | log(no. seqs) | 0.18 | 4.46 | < 0.0001 |
| Lancashire | abiotic | log(no. seqs)  site (WP)  season (Winter)  salinity  soil moisture | 0.2  0.29  0.19  -0.04  0.01 | 4.87  2.8  2.12  -2.19  2.09 | < 0.0001  < 0.01  < 0.05  < 0.05  < 0.05 |
|  | biotic | log(no. seqs)  plant sp. richness  root biomass  rushes | 0.32  0.05  0.02  -0.01 | 7.24  1.93  2.11  -2.75 | < 0.0001  0.053  < 0.05  < 0.01 |
| Overall | abiotic | log(no. seqs)  site (CS)  site (FW)  site (TM)  site (WP)  pH | 0.21  0.28  0.3  0.37  0.67  -0.06 | 7.98  2.18  3.67  4.77  5.21  -1.66 | < 0.0001  < 0.05  < 0.001  < 0.0001  < 0.0001  0.097 |
|  | biotic | log(no. Seqs)  plant sp. richness  root biomass  % cover – herbs/forbs  % cover – shrubs  % cover – grasses  % cover – rushes | 0.3  0.05  0.02  -0.01  -0.002  -0.002  -0.01 | 10.40  2.54  2.56  -3.00  -2.10  -2.24  -2.31 | < 0.0001  < 0.05  < 0.05  < 0.01  < 0.05  < 0.05  < 0.05 |

Table S4. Details of AIC scores from multivariate OTU abundance models with either biotic, or abiotic variables. The number of OTUs with AIC support indicates the number of OTUs for which the difference in AIC between models with abiotic or biotic variables was > 2, indicating sufficient support for one model over the other. OTUs for which the difference in AIC < 2 are included in the “No support” column. ΣAIC is the total AIC across all OTUs for each set of variables. Lower AIC indicates a better overall fit.

| Site/  scale | Number of OTUs with AIC support | | |  | Total AIC (ΣAIC) | |
| --- | --- | --- | --- | --- | --- | --- |
|  | Abiotic variables | Biotic variables | No support |  | Abiotic variables | Biotic variables |
| FW | 263 | 55 | 65 |  | 58,669 | 195,115 |
| AH | 222 | 44 | 54 |  | 51,248 | 206,334 |
| TM | 371 | 84 | 88 |  | 141,271 | 618,692 |
| CS | 291 | 141 | 117 |  | 1,126,393 | 2,107,291 |
| WP | 616 | 251 | 191 |  | 237,236 | 1,642,090 |
| WS | 284 | 73 | 82 |  | 79,791 | 329,615 |
| Essex | 127 | 655 | 129 |  | 847,379 | 1,404,921 |
| Lancashire | 293 | 1,004 | 216 |  | 2,480,136 | 3,267,753 |
| Overall | 1,266 | 498 | 235 |  | 4,353,897 | 14,674,391 |


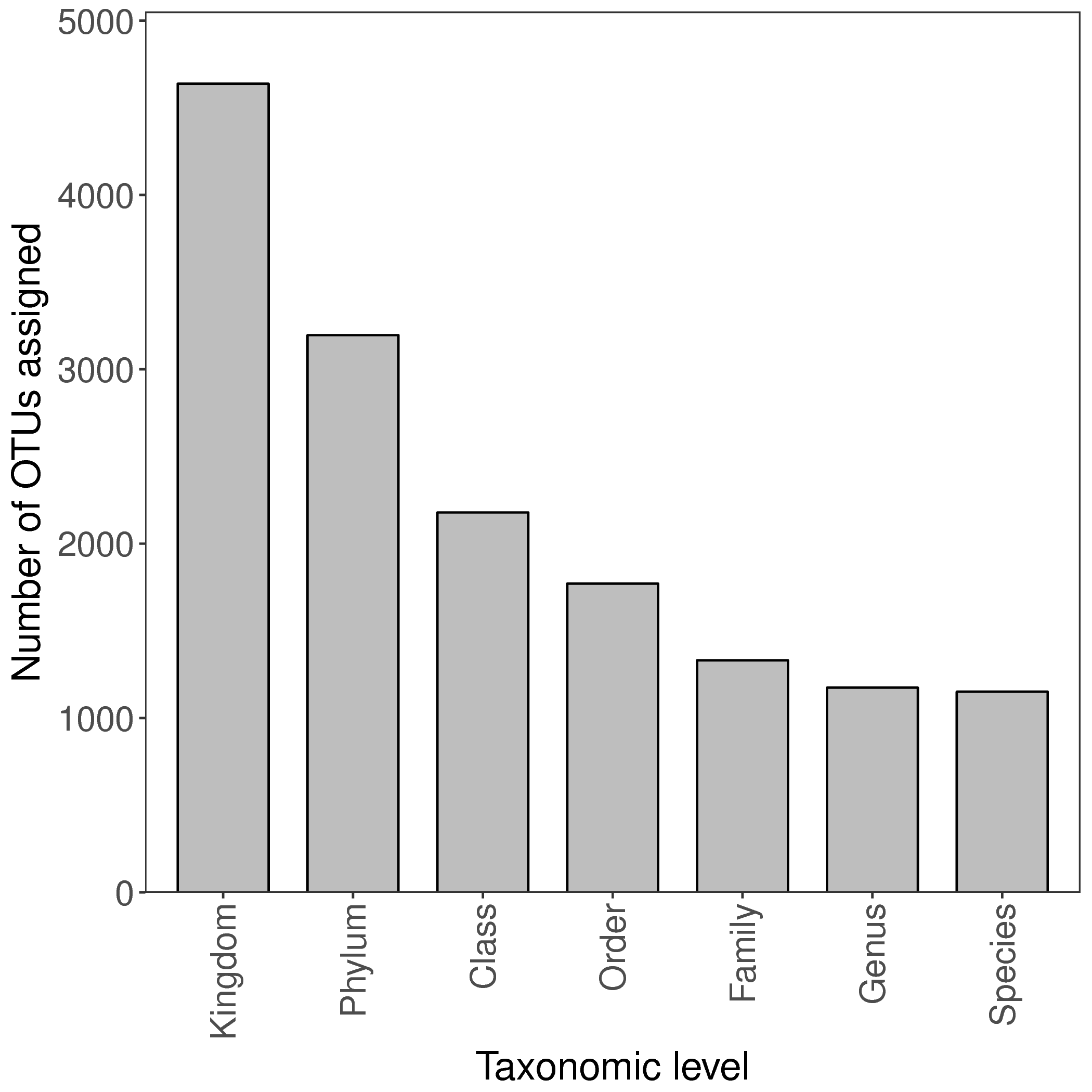
Figure S1. The number of fungal OTUs identified to each taxonomic level using the RDP classifier trained on the UNITE database. Of the 4,641 OTUs, 24.8% could be identified to species level at a 0.7 confidence level.


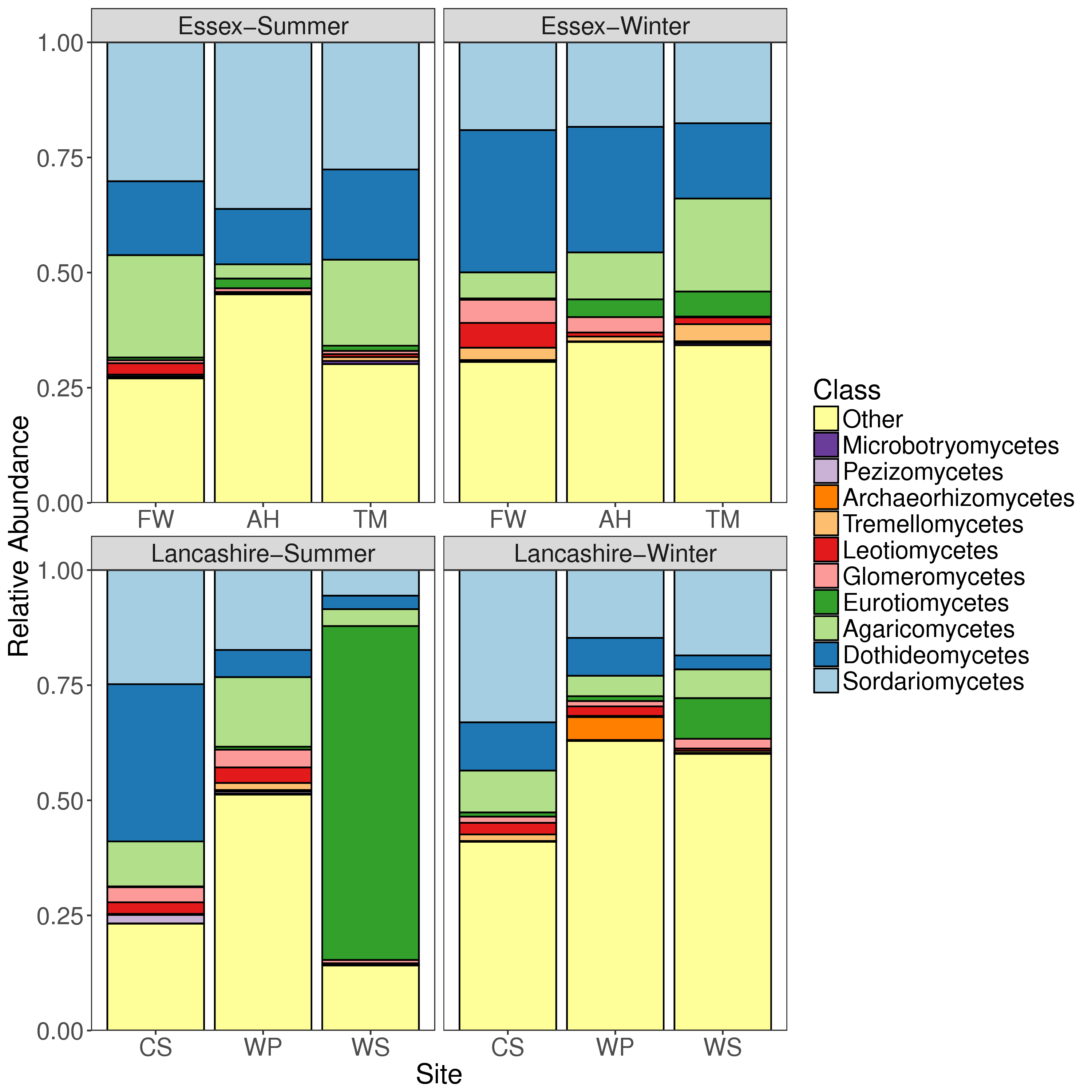
Figure S2. The total relative abundances of the 10 most abundant fungal classes overall, in each site and season. Other less abundant classes were grouped into “other”.


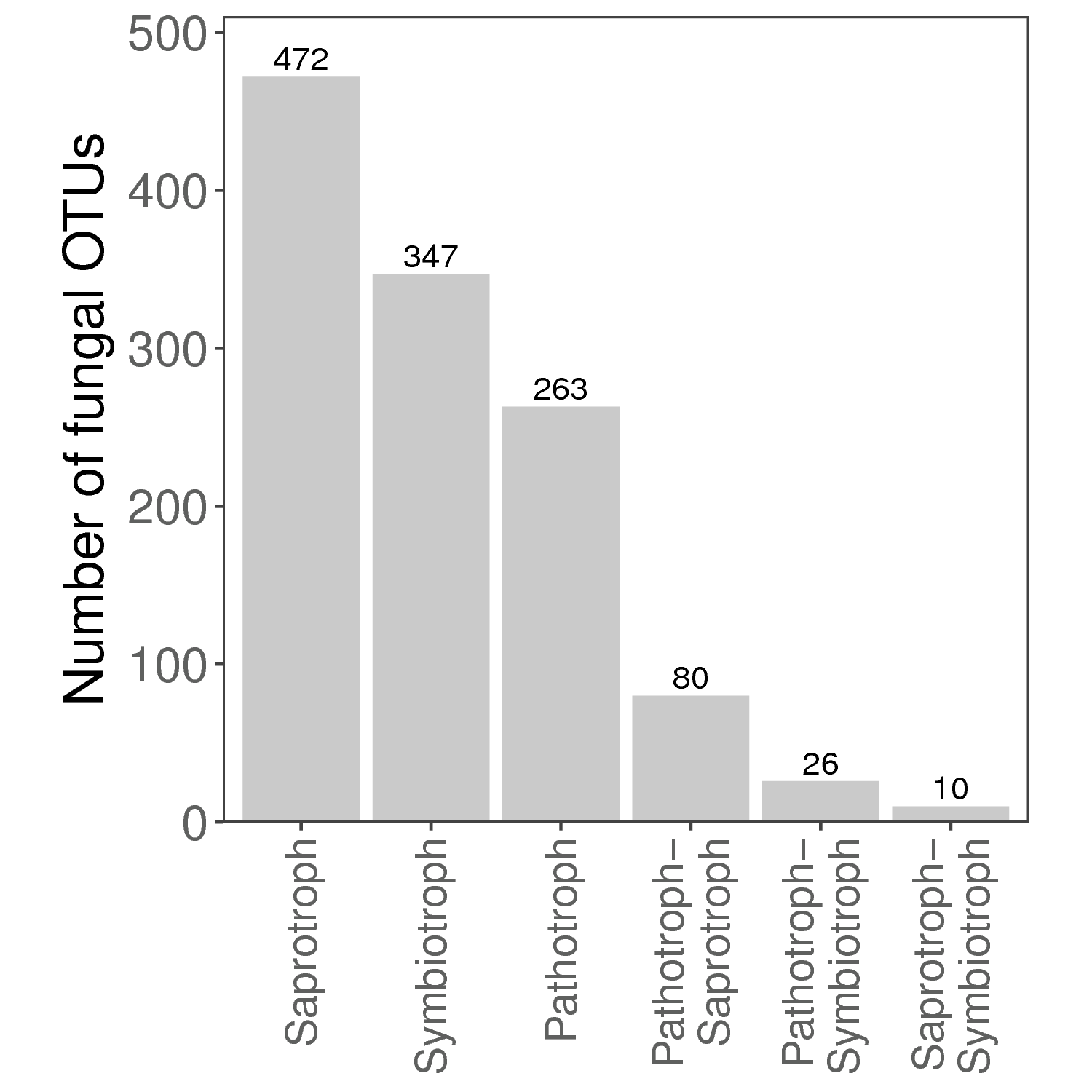


Figure S3. The number of OTUs assigned to trophic groups according to FUNGuild, based on UNITE taxonomic assignments. Fungal OTUs that were not assigned were removed for visualisation purposes. 74.2% of OTUs were not assigned to a trophic group. These OTUs represent OTUs not identified to a high enough taxonomic resolution and/or fungi whose functional traits may not be known/recorded.


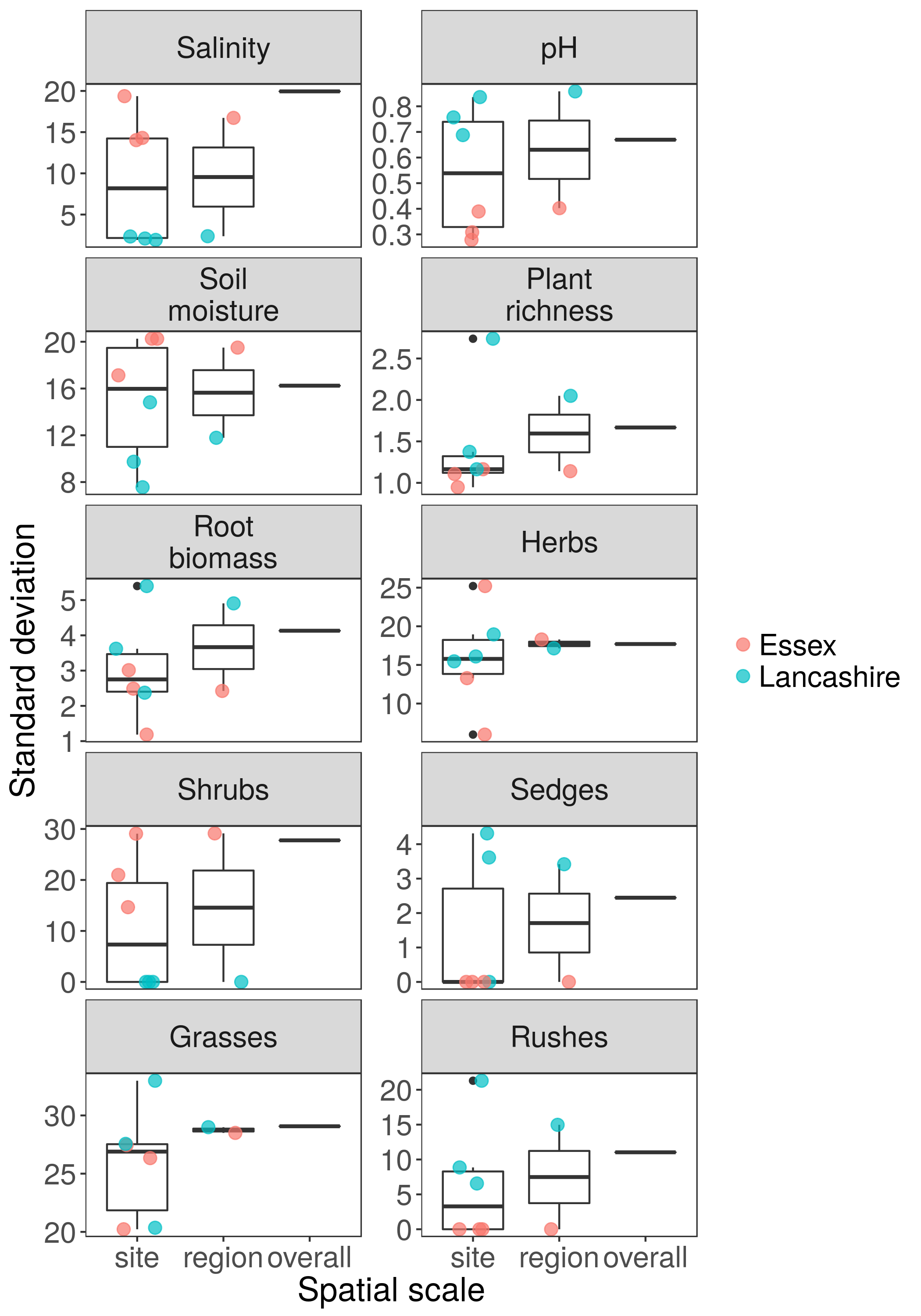
Figure S4. The standard deviation of the mean for each (continuous) predictor variable at each of the spatial scales at which data were analysed. Note that *n* = 6 sites, 2 regions, and 1 overall.
